# Dr. John W. Moffitt, D. D. S.

**Published:** 1914-05-15

**Authors:** 


Dr. John W. Moffitt, D. D. S., died February 27th, 1914, in Harrisburg, Pa., of old age, in his seventy-ninth year.

Dr. John W. Moffitt, a well-known pioneer of modern dentistry, and one of Nature's guileless gentlemen, has crossed to the Great Beyond.

He was the son of the Rev. John J. Moffitt, D. D., and Charlotte Eppley Moffitt, and was born at Orwigsburg, Pa., on June 5th, 1835. Before he had attained his second year his father removed to the (then considered) far West, and settled in Ohio. Early dentists often combined two callings and his father practiced dentistry in addition to attending to his ministerial duties.

Dr. John W. Moffitt received the usual school education of the time, and afterwards attended Bethany College, Va.

He early evinced an aptitude and enthusiasm for dental art. After a period of pupilage with his father, and also with Dr. Samuel Hullein, of Wheeling, Va., (now W. Va.), and thus being instructed in the then highly guarded mysteries of dental art and practice, he opened an office at Cadiz, Ohio, in 1853. Fond remembrance oft carried him back to his first plate, a silver one, because the patient was so satisfied with it, and another which followed shortly after, because the patient counted out fifty, separate, shining one-dollar gold pieces therefor.

In 1855 Dr. Moffitt went on a trip through part of South America and the western part of the United States, and remained quite some time in California. Returning home late in 1857, he married and settled down to practice in Harrisburg, Pa. Since then, until prevented by the state of his health a few years ago, he continued in active practice, except during his service as an Union soldier in the Civil War. When under fire at the battle of Antietem he received orders to report at Hagerstown, Md., as an Assistant Hospital Surgeon, and served in that capacity until honorably discharged in 1864.

Dr. Moffitt was one of our earliest porcelain specialists. Over sixty years ago he carved and baked porcelain blocks and teeth for his own use and that of the profession out of materials dug up and compounded by himself. Afterwards he supervised the manufacture of teeth on a large scale for some of the early manufacturers, personally carving the models and molding, casting and finishing the bronze molds; baking the teeth, and in fact doing everything necessary in tooth manufacture. He always was a strong advocate of high fusing porcelain.

Despite his love for the ceramic art, being an ardent disciple of Izaak Walton, he would leave a continuous gum case to catch a trout.

Ceramics was one of his hobbies. In 1860 he was awarded a patent for non-sectional block work baked on platinum. This he later dedicated to the profession through the Odontographic Society of Philadelphia. Another patent covered a tooth for continuous gum work as now practiced. He also obtained a patent for a tooth for rubber and plastic plates. Quite recently he introduced a method of attaching teeth to the platinum plate without soldering in continuous gum work.

The first bayonet-shaped forceps were made by a Philadelphia firm from a pattern cut out in wood and submitted by him; and many old forceps show his name imprinted thereon.

Dental flasks, adjustable articulators, separators, amalgam alloys, cements, and many other products of his genius have been manufactured, but his modesty and retiring disposition kept him from claiming the honors, and oft-times emoluments received by others therefrom. He was a veritable encyclopedia of means and methods in the preparation of dental necessities, and his fund of information was willingly shared with all.

Obturators and artificial vela were a most successful branch of his practice; and quite recently he made an ingenious and important improvement in the construction of the latter.

He did not often place his ideas in print, although a few articles, chiefly on porcelain subjects, appeared over his signature in dental journals.

He was one of the first delegates to the American Dental Association; and at its meeting at Nashville, Tenn., in 1870, represented the Pennsylvania State Dental Society. He was an officer and charter member of the Pennsylvania State Dental Society, the Lebanon Valley Dental Society, etc., and in fact outlived a number of societies in which he was once prominent. He was also a member of the masonic fraternity.

Dr. Moffitt had a leading part in obtaining from the Pennsylvania Legislature the charter for the Philadelphia Dental College, in which he matriculated in 1864. He, however, did not present himself for examination for his degree until 1888, when he graduated. From the institution of this college he acted as demonstrator in prosthetics and porcelain, with some short intermissions for over forty years; and hundreds of successful practitioners can look back gratefully to his instructions.

He was married in Harrisburg, Pa., on December 20th, 1857, to Harriet R. Wenrick; but his entire family predeceased him. He is survived by a brother, R. H. Moffitt, D. D. S., now retired, and the latters son, Dr. J. J. Moffitt, who is a member of the Pennsylvania State Dental Examining Board.

Interment was in Harrisburg Cemetery on March 2nd, 1914.

